# The Colloid Osmotic Pressure Across the Glycocalyx: Role of Interstitial Fluid Sub-Compartments in Trans-Vascular Fluid Exchange in Skeletal Muscle

**DOI:** 10.3389/fcell.2021.729873

**Published:** 2021-08-12

**Authors:** Fitzroy E. Curry, C. Charles Michel

**Affiliations:** ^1^Department of Physiology and Membrane Biology, School of Medicine, University of California, Davis, Davis, CA, United States; ^2^Department of Bioengineering, Imperial College London, London, United Kingdom

**Keywords:** endothelial glycocalyx, colloid osmotic pressure, capillary permeability, Starling principle, reabsorption, filtration, plasma proteins

## Abstract

The primary purpose of these investigations is to integrate our growing knowledge about the endothelial glycocalyx as a permeability and osmotic barrier into models of trans-vascular fluid exchange in whole organs. We describe changes in the colloid osmotic pressure (COP) difference for plasma proteins across the glycocalyx after an increase or decrease in capillary pressure. The composition of the fluid under the glycocalyx changes in step with capillary pressure whereas the composition of the interstitial fluid takes many hours to adjust to a change in vascular pressure. We use models where the fluid under the glycocalyx mixes with sub-compartments of the interstitial fluid (ISF) whose volumes are defined from the ultrastructure of the inter-endothelial cleft and the histology of the tissue surrounding the capillaries. The initial protein composition in the sub-compartments is that during steady state filtration in the presence of a large pore pathway in parallel with the “small pore” glycocalyx pathway. Changes in the composition depend on the volume of the sub-compartment and the balance of convective and diffusive transport into and out of each sub-compartment. In skeletal muscle the simplest model assumes that the fluid under the glycocalyx mixes directly with a tissue sub-compartment with a volume less than 20% of the total skeletal muscle interstitial fluid volume. The model places limits on trans-vascular flows during transient filtration and reabsorption over periods of 30–60 min. The key assumption in this model is compromised when the resistance to diffusion between the base of the glycocalyx and the tissue sub-compartment accounts for more than 1% of the total resistance to diffusion across the endothelial barrier. It is well established that, in the steady state, there can be no reabsorption in tissue such as skeletal muscle. Our approach extends this idea to demonstrate that transient changes in vascular pressure favoring initial reabsorption from the interstitial fluid of skeletal muscle result in much less fluid exchange than is commonly assumed. Our approach should enable critical evaluations of the empirical models of trans-vascular fluid exchange being used in the clinic that do not account for the hydrostatic and COPs across the glycocalyx.

## Introduction

The glycocalyx is the primary barrier to the movement of plasma proteins between the circulating blood and the tissue fluids. The colloid osmotic pressure (COP) differences across the glycocalyx balances the hydrostatic pressure difference across microvascular walls and holds the blood within the circulation (Levick and Michel, 2010; Michel, 1997; Weinbaum, 1998; Michel et al., 2020). The aim of this paper is to investigate the factors determining this difference in oncotic pressure. Since the plasma lies on one side of the glycocalyx and its value can be estimated under most circumstances, our investigation concerns the factors determining the protein composition of the fluid on the tissue side of the glycocalyx both under steady state conditions and during transients of fluid filtration and reabsorption. The rates of fluid exchange may vary considerably from tissue to tissue with different permeability coefficients of the microvessels and differences in the partitioning of their interstitial fluid (ISF). In this paper, we have used values for permeability coefficients and ISF partitioning for skeletal muscle.

Here, microvascular permeability has been treated in terms of its traditional model where fluid exchange is described in terms of two parallel pathways through microvascular walls: the “small pore” and the “large pore” systems. The “small pores” are the main pathways for exchange of fluid and small hydrophilic solutes and ions. We identify the glycocalyx together with the intercellular clefts of continuous endothelia that act as hydraulic resistances in series as the small pore system (Levick and Michel, 2010; Curry, 2018; Curry et al., 2020; Michel et al., 2020). The large pore system is the separate parallel route that is taken by macromolecules to enter the ISF. While its morphological identity remains uncertain, over-whelming experimental evidence accumulated over the past 65 years supports its existence (e.g., Grotte, 1956; Renkin, 1978; Taylor and Granger, 1984; Rippe and Haraldsson, 1994). Not included at this stage of our analysis is an exclusive water (aquaporin) channel.

The traditional model of microvascular permeability assumed that the effluent of the small and large pores mixed freely in the tissues and the oncotic pressure difference determining transcapillary fluid exchange was that between the plasma oncotic pressure and the average oncotic pressure of the interstitial fluid. Simultaneous measurements of plasma oncotic pressure, average interstitial oncotic pressure and venous or venular interstitial hydrostatic pressures in a range of tissues predicted high levels of fluid filtration inconsistent with measurements of lymph flow (Levick, 1991). In nearly all the tissues showing these discrepancies, the endothelia of the microvessels were continuous (like those of skeletal muscle).

To account for these discrepancies, it was suggested that differences in oncotic pressure across the glycocalyx could differ considerably from the difference estimated from the mean oncotic pressure of the interstitial fluid, particularly in those microvessels with continuous endothelia (Michel, 1997; Weinbaum, 1998; Michel and Curry, 1999). The glycocalyx lies on the luminal side of the endothelia, and in those vessels with continuous endothelia, the fluid filtered through the glycocalyx has to traverse the intercellular cleft including the short breaks in the strand forming the tight intercellular junctions before it reaches the ISF. Previously, steady state fluid exchange across the glycocalyx was modeled in three dimensions to include flow through the intercellular clefts (Hu et al., 2000; Adamson et al., 2004). This model demonstrated that large differences of protein concentration can occur between the general ISF and the sub-glycocalyx space in microvessels with continuous endothelia. This prediction was subsequently supported by experiments in rat mesenteric venules (Adamson et al., 2004).

The sub-glycocalyx space may be considered to be a sub-compartment of the ISF. Small compartments of ISF surrounding pulmonary capillaries had previously been suggested to account for rapid transient fluid exchange following small changes in pulmonary capillary pressures (Hughes et al., 1958; Lunde and Waaler, 1969). Step reductions of hydrostatic pressure in mesenteric capillaries are also followed by very rapid transient absorption of fluid from the tissue. Although these transients are short lived, the volume of the sub-glycocalyx space appears too small to account for them. It has been proposed that a volume of the interstitial fluid, beyond and continuous with, the intercellular clefts of mesenteric capillaries and venules lies between the endothelium and the sleeve of pericytes encasing the vessels. Zhang et al. (2006, 2008) used a simplified 1D form of their 3D model based on the ultrastructure of rat mesenteric venules to account for the time course of transients of fluid exchange following a step change in hydrostatic pressure in these vessels.

Here, we extend the 1D model to describe the transient exchange of water and solutes across the glycocalyx in skeletal muscle, where the transients of fluid exchange are much slower, continuing for more than 30–60 min. We use vascular permeability characteristics and anatomical features of the ISF of muscle to guide our model development.

## Materials and Methods

### Model Development

**FIGURE 1:**
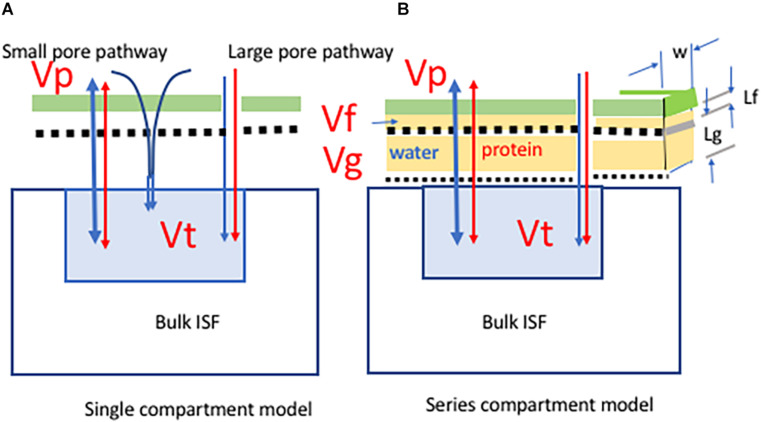
Two models of water (blue) and protein (red) exchange from the plasma *Vp* to a sub-compartment of the interstitial fluid space *Vt*. In both models the restriction to movement of plasma proteins is determined by the fiber size and fiber spacing in the glycocalyx (green) and the glycocalyx is the primary barrier to the movement of protein from circulating plasma to the interstitial fluid. The fluid leaving the glycocalyx is funneled through breaks in the junction strands in the inter-endothelial cell junctions. A small number of large pores are in parallel with the glycocalyx-junction pathway. The colloid osmotic pressure difference determining water exchange is between the plasma and the fluid under the glycocalyx. The model on the panel **(A)** (single tissue compartment model) assumes the fluid leaving the glycocalyx mixes with a single tissue sub-compartment (volume *Vt*) formed from a portion of the ISF that is in close proximity to microvessels. The model on the panel **(B)** (series compartment model) has two additional sub-compartments. *Vf* is within the inter-endothelial cell cleft and directly under the glycocalyx. *Vg* lies within the inter-endothelial junction and between the breaks in the junctional strands and the tissue sub-compartment. The volumes of these sub-compartments are determined from published details of the ultrastructure of the intercellular junctions and the position of the junction strand as indicated in panel **(B)**. In both models the PS product of the glycocalyx barrier is PdS1 and of the large pore pathway is PdS4. In the series compartment model PdS2 accounts for the barrier between *Vf* and *Vg* and PdS3 accounts for the barrier between *Vg* and *Vt*. Tables 1, 2 describe the model parameters in detail.

Two models are shown in Figure 1. The simplest model (Figure 1A, the tissue sub-compartment model) assumes the fluid leaving the glycocalyx mixes with a single tissue sub-compartment (volume Vt) formed from a portion of the ISF in close proximity to microvessels. We describe the anatomical equivalent of this tissue sub-compartment for muscle as part of the development of this model. The second model (Figure 1B, the series compartment model) extends this approach to include two additional sub-compartments, *Vf* directly under the glycocalyx (the glycocalyx sub-compartment), and Vg created by the arrangement of the junctional strands in the intercellular cleft.

Previously, steady state fluid exchange across the glycocalyx on the luminal surface of the endothelium and the intercellular cleft was modelled using estimates of (i) the average distance between the base of the glycocalyx and the barrier formed by the tight junctions within the cleft *Lf* and (ii) the distance from infrequent breaks in this barrier and the exit from the intercellular cleft to the tissue sub compartment *Lg* (Hu et al., 2000; Adamson et al., 2004). Here, we estimate the volumes of sub-compartment that lie directly beneath the glycocalyx and proximal to the array of junctional strands within the intercellular cleft as *Lf.W* × *Lj*/2 and the volume of the second sub-compartment between breaks in the junction stand and the cleft exit as *Lg.W.Lj*/2 where *W* is the mean width of the intercellular cleft taking into account that the cleft tends to be wider at the cleft entrance and exit and *Lj* is the endothelial cleft length per unit area taking into account that on average two endothelial cells share a common line of contact (Figure 1B).

Table 1 lists the parameters in the models and Table 2 their assigned values. Because we describe transvascular exchange representative of skeletal muscle, we have scaled all parameters to a mass of 40 kg of muscle in a lean 70 kg human subject.

**TABLE 1:** Symbols, abbreviations, and subscripts.

*C*, concentration mg/ml
COP, colloid osmotic pressure mmHg millimeters of mercury
ISF, interstitial fluid mls
*Js*, solute flux in mg/min
*Jv*, water flux in ml/min
*L*, distances within the intercellular cleft
LpS, capillary filtration coefficient ml/min/mmHg
*P*, pressure in mmHg
*Pd*, is solute permeability coefficient cm/s
*PS*, is permeability surface area product ml/min
Pe, Peclet number dimensionless
*S*, surface in cm^2^
*T*, time in seconds or minutes
*V*, is volume mls
*w*, width of inter cellular cleft
*Z*, dimensionless term = Pe/(exp(Pe) − 1)
*Π*, colloid osmotic pressure (COP) mmHg
σ, osmotic reflection coefficient
Subscripts
*p*, *f*, *g*, *t* in plasma, subglycocalyx, cleft, and tissue
1, 2, 3, 4 permeability coefficients in the barriers across glycocalyx, glycocalyx to cleft, cleft to tissue, and large pore, respectively

**TABLE 2:** Model parameters.

*C*_*p*_, plasma protein concentration 69 mg/ml, COP = 25 mmHg
*L*_*f*_, 100 nm (Hu et al., 2000; Adamson et al., 2004)
*L*_*g*_, 600 nm (Hu et al., 2000; Adamson et al., 2004)
*L*_*j*_, 1,000 cm/cm^2^ (Adamson, 1993)
LpS_1_, 2 ml/(min mmHg) from Michel and Moyses (1985)
LpS_4_, 0.02 ml/(min mmHg) calculated using Taylor and Granger (1984)*
P_*d*_S_1_, 0.4 ml/min (Renkin and Tucker, 1998)
P_*d*_S_2_, is 5.6 ml/min Estimated from Zhang et al. (2006)*
P_*d*_S_3_, 50 ml/min Estimated for albumin diffusion in ISF*
*S*, surface 7,000 cm^2^/100 g (Pappenheimer et al., 1951)
*V*_*f*_, 0.05 ml Calculated as *Lf.w.Lj*/2
*V*_*g*_, 0.3 ml Calculated as *Lg.w.Lj*/2
*V*_*t*_, 1,000 ml Estimated as 1/6 of ISF volume*
*w*, 20 nm (Adamson and Michel, 1993; Adamson et al., 2004)
σ_1_, 0.95 (Michel, 1984)
σ_2_, σ_4_, 0.2 estimated for albumin in slit, width 20 nm (Curry, 1984)
σ_3_, 0 estimated for albumin in ISF

*See text for further details.

### The Volume of Tissue Sub-Compartment

Clues to interstitial fluid distribution in skeletal muscle are provided by the arrangement of three layers of its connective tissue. The muscle cells (or fibers) are long thin cylinders and each is surrounded by a thin layer of connective tissue called the endomysium. The cells are collected into bundles, the number of cells per bundle varying with the function of the muscle: small number of fibers – for fine movements as in external eye muscles; large numbers of fibers per bundle in muscles contributing to strength. The bundles are surrounded by a layer of connective tissue called the perimysium. Bundles making up the entire muscle are surrounded by an outer layer of connective tissue called the epimysium (Csapo et al., 2020; Wang et al., 2020).

**FIGURE 2:**
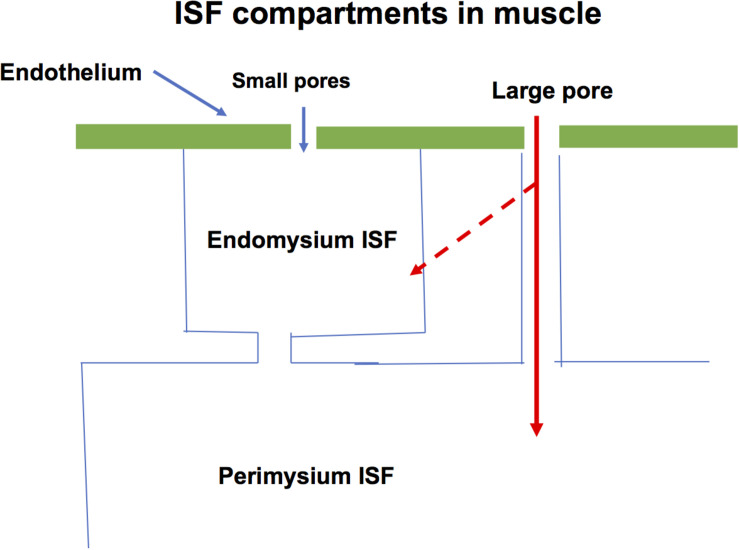
A specific form of Model in Figure 1A used to describe water and solute exchange in skeletal muscle is shown. The endomysium is adjacent to the capillaries and is estimated to occupy one sixth of the interstitial fluid space. The bulk of the ISF is in the perimysium. During the transient exchanges investigated in this paper it is assumed that exchange across both the glycocalyx and large pore pathways mixes into the endomysium ISF (broken arrow).

The endomysium surrounding each multi-nuclear muscle cell carries three or more capillaries supplying the adjacent muscle cells. Arterioles and venules are found in the perimysium with occasional branches running perpendicular and usually together to supply and drain the capillaries of the endomysium. If we assume the large pores responsible for macromolecular exchange between blood and interstitial fluid are located in the venules, the ISF in the endomysium would form a sub-compartment adjacent to the capillaries. To estimate the volume of this sub-compartment, we assume it surrounds each muscle cell with a thickness of 0.5 μm. If the average diameter of the muscle cell is 80 μm, the endomysial fluid would represent just less than 2.5% of the cross-section area of cell plus ISF sub-compartment. If the total volume of ISF is 15% of the muscle mass, the endomysial fluid is one sixth of the total ISF. In a lean 70 kg man, 40 kg of which is skeletal muscle, this would represent one liter of fluid. It is possible that this volume is split into short stretches of 100–300 μm along the length of the muscle cells by the transverse branches of arterioles and venules as they run to and from the capillaries. But the endomysial fluid surrounding one cell is continuous with fluid surrounding adjacent cells increasing the volume of the compartment as a whole. In all the calculations below, we used 1,000 ml for the tissue sub-compartment. Figure 2 shows a model for muscle.

One form of the model assumes the large pores mix with the smaller endomysium compartment, the other assumes large pores mix only with the large perimysium compartment. The steady state relations do not require a distinction between exchange into the tissue sub-compartments. However, for modeling of transient exchange we examined only the limiting case where all large pore exchange occurs into the endomysial sub-compartment.

### Volume of the Sub-Compartment Under the Glycocalyx and in the Endothelial Cleft Between the Glycocalyx and the Tissue Compartment

To scale the length of the line of junction (*Lj*) 1,000 cm/cm^2^ for a 40 kg muscle mass we used 7,000 cm^2^/100 gm as an estimate of surface area (Pappenheimer et al., 1951). This value is for fully dilated cat and dog hindlimb. The corresponding volume for a region within the intercellular cleft 100 nm deep, 20 nm wide under the glycocalyx is 0.056 mls. On one hand this estimate would be lower when the microvasculature was not fully dilated. On the other hand, the cleft is often seen to be wider than 20 nm directly under the glycocalyx at the cleft entrance. We used 0.05 mls for *Vf*. The cleft volume was set at 0.3 mls based on a ratio of *Lg/Lf* of close to 6 (Figure 1B; Adamson et al., 2004).

### Permeability Parameters

The published clearances of albumin in rodent skeletal muscle, 0.1–0.12 μL/min per gram of dry tissue weight, scale to 0.48–0.72 ml/min for the 40 kg muscle mass assuming a wet to dry weight ratio of 5 (Renkin and Tucker, 1998). We used the value of 0.5 ml/min corresponding to an albumin permeability coefficient of 1 × 10^–8^ cm/s. A PS product of 0.5 ml/min accounts for both large pore and small pore diffusive pathways. We partitioned the PS product as 0.4 ml/min for the small pore pathway glycocalyx and 0.1 ml/min for large pores based on estimates of their size and frequency in skeletal muscle (Taylor and Granger, 1984).

We used the results from the 1D model of Zhang et al. (2006) to assign a preliminary PS product to the barrier beneath the glycocalyx sub-compartment. Under conditions where there was no net flow across the glycocalyx in the 1D model, the glycocalyx accounted for 93% of the concentration difference between plasma and tissue. This corresponded to a ratio of diffusion resistance in the glycocalyx to the diffusion resistance beneath the glycocalyx of 14:1. With a glycocalyx PS of 0.4 ml/min, a similar distribution of diffusion resistances for muscle gives a PS product of 14 × 0.4 ml/min or 5.6 ml/min for the barrier between the glycocalyx and the cleft. This estimate takes into account the extra resistance as solute flows are funneled into infrequent breaks in the junction strand. It is also assumes that the PS product for albumin in muscle is fivefold to 10 fold less than values in mesentery because the more complex arrangement of the junction strand in the endothelial barrier muscle reduces the effective size and frequency of breaks (Wissig and Williams, 1978; Bundgaard, 1984). We also tested other values of PS that accounted for more resistance in the junction strands (PS = 3 ml/min where the strands accounted for 10% of the resistance as well as PS = 12 and 24 ml/min where the strands accounted for resistances of 4 and 1% the resistance to solute diffusion, respectively.

We set the PS product for diffusion from the cleft into the issue compartment as 50 ml/min corresponding to a mean diffusion distance as high as 150 μm based on diffusion distances that could range from a minimum of 12 μm for direct mixing with a 1,000 ml volume, to mixing with isolated segments 300 μm apart. All model results were relatively insensitive to the value of this parameter.

### Capillary Filtration Coefficient LpS

Michel and Moyses (1985) measured the swelling of human forearm with increments of venous pressure in healthy human subjects as 3.2–4 × 10^–3^ ml/(min mmHg 100 g) after allowances had been made for bone content of the forearm. This value was close to earlier estimates by Landis and Gibbon (1933). The value increased to the order of 5 × 10^–3^ ml/(min mmHg 100 g) as a reasonable approximation to the swelling due to changes in capillary pressure (rather than venous pressure) based on values of the ratio of arteriolar to venular resistance to blood flow in animal and human experiments (Pappenheimer and Soto-Rivera, 1948; Levick and Michel, 1978). The value 5 × 10^–3^ ml/(min mmHg 100 g) leads to a figure of 2 ml/(min mmHg) for 40 kg muscle.

The reflection coefficient of the glycocalyx was set at 0.95 (Michel, 1984). The solvent drag reflection coefficient in the large pore and lateral cleft was 0.2 based on a cleft width of 20 nm (Curry, 1984). Hydrostatic pressures below the glycocalyx, in the intercellular cleft and tissue are assumed close to zero but a finite pressure gradient is required to drive flow from the tissue toward the glycocalyx sub-compartment.

### Steady State Relations for the Single Tissue Compartment Model

The relations for the single tissue model are given below, followed by relations for the series-compartment model. To calculate the concentration difference of protein across the glycocalyx, the small pore pathway is treated as membranes in series. For each membrane, the diffusive permeability-surface area product is PS and the solvent drag reflection coefficient is σ. For the protein flux from plasma to a tissue sub-compartment, the solvent drag component of the protein flux is *Jv* (1 − σ) *Cp* where *Jv* is the water flux and *Cp* is the plasma protein concentration. The diffusive component of the protein flux between the plasma and a tissue sub-compartment (protein concentration *Ct*) is PS (*Cp* − *Ct*).*z* where *z* is Pe/(exp(Pe) − 1) with Pe, the Peclet number, given by (*Jv*(1 − σ)/PS) (Curry, 1984). In the following, subscripts 1 and 4 refer to the membrane barriers in the glycocalyx and the large pores, respectively. Subscripts2 and 3 are used in the series model to refer to the additional membrane barriers in the intercellular cleft.

For Steady state exchange across the glycocalyx and large pores in the single tissue compartment,

(1) J⁢v⁢Total⁢C⁢t=J⁢s⁢1+J⁢v⁢4

*Jv*_*total*_ is the water flow across both pathways in ml/min, *Jv*_*total*_ = *Jv*1 + *Jv*4. *Ct* is the concentration in the tissue sub-compartment *Js*1 is small pore flux and *Jv*4 is large pore flux.

(2) J⁢s⁢1=J⁢v⁢1⋅(1-σ⁢1)⋅C⁢p+P⁢d⁢S⁢1⋅(C⁢p-C⁢t)⁢z⁢1

and

(3) J⁢s⁢4=J⁢v⁢4⋅(1-σ⁢2)⋅C⁢p+P⁢d⁢S⁢4⋅(C⁢p-C⁢t)⁢z⁢4

Substituting in Eq. 1:

(4) $$C\,t=C\,p\cdot \frac{\left\{J\,v\,1\,\left(1-\sigma \,1\right)+P\,d\,S\,1\,z\,1+J\,v\,4\,\left(1-\sigma \,4\right)+P\,d\,S\,4\,z\,4\right\}}{\left\{\left(J\,v\,1+J\,v\,4\right)+P\,d\,S\,4\,z\,4+P\,d\,s\,1\,z\,1\right\}}$$

The fluid exchange across the glycocalyx is described by the Starling principle,

(5) J⁢v⁢1=L⁢p⁢S⁢1⋅{(P⁢p-P⁢t)-σ⁢1⁢(π⁢p-π⁢t)}

where *Pp* and *Pt* are the pressures in the capillary and the tissue sub-compartment and π*p* and π*t* are the corresponding COPs calculated from the plasma protein concentrations at 37°C by the relation,

(6) $$\pi =2.1\,C+0.16\,C^{2}+0.009\,C^{3}.\ldots .$$

(Landis and Pappenheimer, 1963)

*C* is mg/ml. The relation is used to calculate COP in all compartments. The relation is strictly valid only for fluid with the protein concentrations of circulating plasma. In this paper the same relation is used to convert tissue protein concentrations to COPs. This ignores the fact that COP for ISF fluid should be calculated from a relation closer to that expected for albumin. The composition of the fluid under the glycocalyx and tissue differs from the plasma because larger plasma proteins such as IgG are retained in the plasma and the unit mass of tissue protein contains more albumin than a corresponding mass of plasma protein. This difference is ignored in the present calculations because the relation corresponding to Eq. 6 for ISF fluid has not been reported.

The fluid exchange across the large pores is:

(7) J⁢v⁢4=L⁢p⁢S⁢4⋅{(P⁢p-P⁢t)-σ⁢4⁢(π⁢p-π⁢t)}

It is assumed that tissue pressure is zero.

The key assumption in the single tissue model is that the effluent leaving the base of the glycocalyx mixes instantly with the tissue compartment contents. To enable comparison of this model with the series compartment model where there are barriers to the exchanges between the effluent behind the glycocalyx and the tissue sub-compartment, it is useful to note that a well-mixed volume is one where diffusion gradients do not exist. With this assumption for the single compartment model, the flux of protein carried away from the base of the glycocalyx and equal to the flux across the glycocalyx is written as,

(8) J⁢s⁢1=J⁢v⁢1⋅C⁢f

where *Cf* is equal to *Js*1/*Jv*1 in Eq. 2.

(9) C⁢f=C⁢p⋅(1-σ⁢1)/{1-σ⋅e⁢x⁢p⁢(-P⁢e)}

*Cf* can be understood as the concentration of effluent from beneath the glycocalyx fluid that would be measured if this effluent did not mix directly and immediately with the contents of the ISF. Furthermore, it is noted that Eq. 8 has the form of a convection dominated equation, not because *Jv*1 is high but because the well mixed assumption is equivalent to the assumption that convection dominates at all values of *Jv*.

The steady state relation between *Jv*_*total*_ and pressure was constructed by substituting *Jv*.*Cf* for *Js*1 in Eq. 2, so that,

(10) $$Ct=Cf\cdot \left(\frac{J\,v\,1}{\left\{J\,v\,1+\left(J\,v\,4\right)\right\}}\right)+\{Jv4\left(1-\sigma 2\right)\cdot Cp+PdS4z4\left(Cp-Ct\right)\}/(Jv1+Jv4)$$

The following iterative steps were used to construct the steady state relation between *Jv* and pressure. We note that for a given value of *Jv*1, the values of *Pe*1 and *z*1 in Eqs. 2 and 3 are known for assigned values of PS and σ.

Step 1: For a given *Jv*1, a first approximation for tissue concentration is calculated from Eq. 10 neglecting the large pore contribution.

Step 2: A capillary pressure is then calculated from Eq. 5. This capillary pressure acts across both small pores and large pores.

Step 3: *Jv*4 was calculated from Eq. 7 using the estimated Pc in step 2 and the COP for the initial estimate of *Ct*.

Step 4: The new *Jv*4 and *Pc* are used to calculate a new *Ct* from Eq. 10. Successive iterations quickly converged to consistent estimates of *Ct*, and *Jv*1, and *Jv*4 at each capillary pressure. All calculations are carried out in Excel worksheets.

### Transient Changes in Fluid Exchange

The time course of changes in protein concentration in the tissue sub-compartment is calculated by numerical solution of the differential equation describing the mass balance relation:

*Vt*(*T*)d*Ct*/d*T* = *Js*1(*T*) + *Js*4(*T*)

The calculation of the concentration *Ct* as a function of time *T* was carried out with time steps (Δ*T*) of 1 min in an Excel worksheet: *Ct* at time *T* + 1 relative to *Ct* at time *T*:

(11) C⁢t⁢(T+1)={V⁢t⁢(T)⋅C⁢t⁢(T)+J⁢s⁢1⁢(T)+J⁢s⁢4⁢(T)}/Vt⁢(T)

Here, *Js*1 and *Js*4 are calculated from Eqs. 3 and 8 with the values of *Cp* and *Ct* at time *T*.

### Series Compartment Model

The same approach as for the single compartment is used. The overall steady state relation is the same as text Eq. 1 but *Js*1 is the flux from the sub-compartment in the cleft (*Cg*) into the tissue compartment.

(12) J⁢v⁢1⁢C⁢g⁢(1-σ⁢3)⁢C⁢g+P⁢d⁢S⁢3⁢z⁢3⁢(C⁢g-C⁢t) +J⁢v⁢4⁢(1-σ⁢4)⁢C⁢p+P⁢d⁢S⁢4⁢z⁢4⁢(C⁢p-C⁢t)=C⁢t⁢(J⁢v⁢1+J⁢v⁢4)

Equation 12 can be written as

(13) C⁢t=A.C⁢g+B

Where

(14) A=Cp[Jv1(1-σ1)+PdS3z3]/{(Jv1+Jv4)+PdS3z3+PdS4z4}

and

(15) B=Cp[Jv4(1-σ4)+PdS4z4]/{(Jv1+Jv4)+Pds3z3+Pds4z4}

Continuity requires the flux into the cleft compartment from the sub-compartment under the glycocalyx to equal the flux out of the cleft compartment into the tissue,

(16) J⁢v⁢1⁢(1-σ⁢2)⁢C⁢f+P⁢d⁢S⁢2⁢z⁢2⁢(C⁢f-C⁢g)=J⁢v⁢1⋅C⁢g⁢(1-σ⁢3)+P⁢d⁢S⁢3⁢z⁢3⁢(C⁢g-C⁢t)

Substitute for *Ct* from equation,

(17) C⁢g=C.C⁢f+D

Where

(18) C=[Jv1(1-σ2)+PdS2z2]/{Jv1+PdS2z2+PdS3(1-A)}

And

(19) D=P⁢d⁢S⁢3⁢z⁢3⁢B/{J⁢v⁢1+P⁢d⁢s⁢2⁢z⁢2+P⁢d⁢s⁢3⁢z⁢3⁢(1-A)}

Similarly, the flux from plasma compartment across the glycocalyx equals flux out of the sub-compartment under the glycocalyx.

(20) J⁢v⁢1⁢(1-σ⁢1)⁢C⁢p+P⁢d⁢S⁢1⁢z⁢1⁢(C⁢p-C⁢f)=J⁢v⁢1⁢C⁢g⁢(1-σ⁢2)+P⁢d⁢S⁢3⁢z⁢3⁢(C⁢g-C⁢t)

Substitution for *Cg* gives *Cf* as a function of *Cp*.

The steady state relation between *Jv*_*total*_ and capillary pressure is calculated from an iterative process as described for the single compartment model. The transient equations are calculated from mass balances over each compartment as in Eq. 11. Values of *Cf*, *Cg*, and *Ct* were calculated using the same numerical method as used for the single tissue compartment model. As explained in the section “Discussion,” time steps were reduced to 0.1 s to account for rapid changes in concentration in the very small volumes of the glycocalyx and cleft sub-compartments.

## Results

### Steady State Relation Between *Jv*_*total*_ and Pressure With and Without Large Pore

**FIGURE 3:**
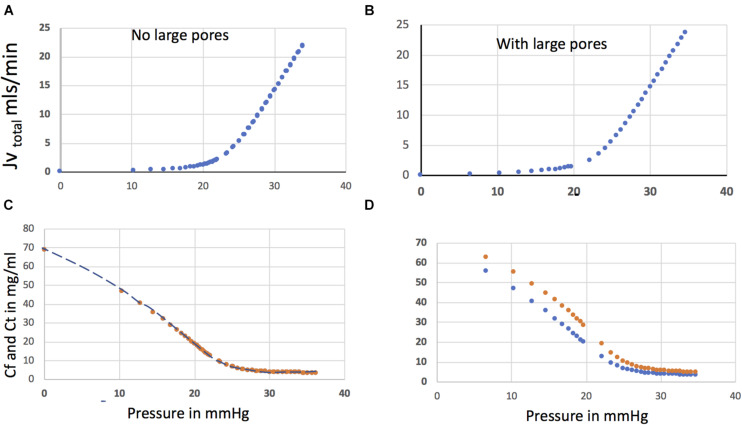
Steady state relations between *Jv*Total (*Jv*1 + *Jv*4) and Pressure [top panels **(A)** and **(B)**] with and without large pores. The lower panels of Figure 3 show the concentration of plasma protein below the glycocalyx (*Cf*) (blue) and in the interstitial fluid (*Ct*) (red) as a function of capillary pressure with and without the presence of large pores. These relations are independent of the size of the ISF as the steady state ISF concentration is determined only by the relation *Ct* = *Js Total*/*Jv Total*. Many hours are required to establish steady state. Panel **(C)** shows that in the absence of large pores the ISF fluid has the composition of the fluid leaving the base of the glycocalyx. When the flux through large pores also mixes with the ISF (**D**) there is a difference in concentration between the fluid leaving the base of the glycocalyx and the tissue fluid.

Figure 3 shows steady state relations for *Jv*_*total*_ (*Jv*1 + *Jv*4) versus Pressure (Top Panels) with and without large pores as a function of presssure. The hockey stick shaped relation between total water flux and capillary pressure is raised up and moved slightly to the left by the presence of the large pores. The LpS of the large pore is 1% of the small pore, glycocalyx pathway, but at a pressure of 13 mmHg large pores carry 40% of the protein flux as solvent drag. As pressure increases the fraction of total flow through the large pores reduces to 10% or less above *P* = 25 mmHg.

The lower panel of Figure 3 shows the concentration of plasma protein below the glycocalyx (*Cf*) and in the tissue (*Ct*) as a function of pressure. When there are no large pores, the steady state tissue concentration is determined solely by the transport across the glycocalyx. In the presence of large pores, Figure 3D shows the difference between the concentration in the tissue and that leaving the glycocalyx before mixing with the tissue. At 20 mmHg capillary pressure, the concentration of plasma protein in the tissue is 27 mg/ml (COP = 7.3 mmHg when Eq. 6 is used). This concentration is 7 mg/ml greater than that fluid leaving the glycocalyx 20 mg/mg (COP 4.9 mmHg), which in this model mixes instantly with the tissue. A key part of the Revised Starling principle is demonstrated by these observations. The effective osmotic pressure difference that opposes filtration across the glycocalyx is larger than that predicted from the difference between plasma and the average interstitial plasma protein concentration. Specifically, with a glycocalyx reflection coefficient of 0.95 and plasma COPs of the effective COP opposing filtration across the glycocalyx (19 mm Hg) is 2.1 mmHg larger than would be calculated from the plasma to average interstitial protein concentration difference. This difference is small but an important general observation is that for capillary pressures in the range 13–25 mmHg, steady state fluid exchange between plasma and tissue is determined by small differences in hydrostatic pressure and osmotic pressures, because COP and capillary pressure are closely balanced. The following examples demonstrate the importance of understanding this balance across the glycocalyx after a change in pressure from a well-established steady state.

### Transient Filtration and Reabsorption for the Single Tissue Compartment Model With Large Pore and Endomysial Compartment

Because the model with mixing of the effluent below the glycocalyx directly into a tissue compartment describes the limiting case where there are no additional diffusion barriers between the glycocalyx and the tissue sub-compartment, the results are presented first. To enable comparison with the series compartment model where diffusion barriers are present, the single tissue compartment model is used to evaluate the filtration transient when pressure is reduced from 20 to 17 mmHg and the reabsorption transient when pressure is reduced from 20 to 13 mmHg. These two examples cover the same range of pressure examined in the series-compartment model (*P* = 20–13 mmHg).

#### Transient Filtration

The concentration of plasma protein in the tissue during steady state filtration at *P* = 20 is 27 mg/ml; COP = 7.3 mmHg) as in Figure 3D. When the pressure is suddenly reduced to 17 mmHg and this tissue protein concentration is used as a measure of the protein concentration under the glycocalyx, the effective COP opposing filtration is 16.8 mmHg, calculated as 0.95(25–7.3) mmHg. The corresponding initial filtration rate across the glycocalyx is 0.4 ml/min (Eq. 5). This value is much lower than the filtration rate at a steady state with pressure of 17 mmHg. At steady state the tissue concentration is 37 mg/ml and the colloid pressure opposing filtration is reduced to 12.8 mm Hg, however, this low filtration rate right after pressure is reduced to 17 mmHg would continue for some time because tissue concentration will increase slowly toward the steady state because protein is added to the tissue volume only by exchange through large pores.

One estimate of the half time for fluid filtered through both the large pore and the glycocalyx is the ratio of the large pore filtration rate to tissue volume. The initial large pore filtration rate is 0.38 ml/min (Eq. 7 with reflection coefficient as 0.2). For a tissue volume of 1,000 ml, the corresponding time constant is 0.78 × 10^–3^ min^–1^ and the half time for exchange exceeds 1,000 min. It follows that over a 30-min period early in the transient, the model predicts a loss of less than 25 mls from the plasma volume.

#### Reabsorption With Single Tissue Compartment

During reabsorption tissue fluid enters the junction clefts behind the glycocalyx when reabsorption begins across the glycocalyx. The rate plasma protein accumulates in the intercellular cleft beneath the glycocalyx is a race between convective transport carrying solute into the cleft and diffusion of the accumulated solute back toward the tissue. The assumption that the fluid under the glycocalyx mixes with the tissue fluid means that the concentration under the glycocalyx cannot exceed the tissue concentration. This is equivalent to assuming that back diffusion from the base of the glycocalyx to the tissue sub-compartment ensures that the solute concentration at the back of the glycocalyx has a value equal to the tissue concentration. The model therefore describes the maximum COP difference between plasma and the tissue under the glycocalyx and the corresponding maximum rate of reabsorption after pressure is reduced from a steady state.

The initial effective COP across the glycocalyx at a pressure of 20 mmHg, determined by steady state filtration into the tissue sub-compartment, is the same as at the beginning of the filtration transient described above. But when pressure is reduced to 13 mmHg, the balance of hydrostatic and COP is −3.8 mmHg and favors reabsorption. The reabsorption rate across the glycocalyx is −7.5 ml/min. This rate slows as plasma proteins are diluted and solute accumulates in the tissue by exchange through large pores. These reabsorption rates differ significantly from the steady states at *P* = 13 mmHg which is slow filtration at a rate of less than 0.5 ml/min (Figure 3B).

**FIGURE 4:**
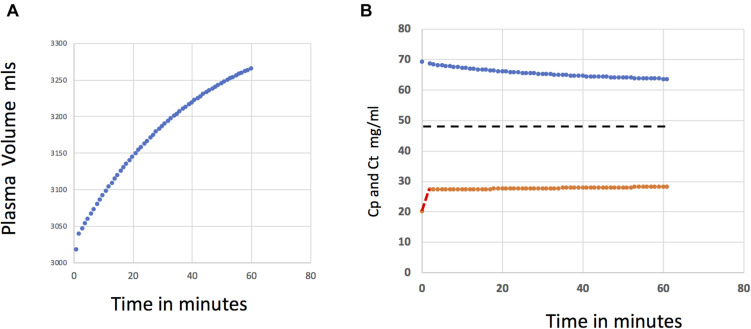
**(A)** Plasma volume is shown as a function of time during reabsorption after capillary pressure reduced from 20 to 13 mmHg. The rate of reabsorption predicted using models Model 1a scaled for skeletal muscle (Figure 2) is a maximum because protein accumulation in the fluid behind the glycocalyx cannot exceed with concentration of the ISF in the tissue sub-compartment (volume 1,000 ml). **(B)** The protein concentrations in in vascular space (*Cp*) (blue) is reduced as reabsorbed fluid is added to the vascular volume. The tissue protein concentration *Ct* increases slowly as protein flux through large pores accumulates in the tissue sub-compartment. It takes many hours for the protein concentration in the tissue compartment to reach the steady state concentration for pressure of 13 mmHg (broken black line).

Figure 4A shows the reabsorption transient using the single tissue compartment model of Figure 2. Tissue concentrations calculated from a mass balance over the tissue compartment (Eq. 11) are in Figure 4B. Protein concentration in the tissue sub-compartment slowly approaches the steady state value of 47 mg/ml (Broken line) corresponding to a condition of steady state filtration at a pressure of 13 in Figure 3D. Plasma protein concentration falls with dilution by reabsorbed fluid.

From Figure 4A, 180 ml are reabsorbed after 30 min and a maximum of 270 ml reabsorbed after 60 min. For the assigned model parameters, this rate of reabsorption is the largest expected for a tissue mass of 40 kg of muscle in a human subject.

Two conclusions follow from investigations using the single tissue compartment. First, a more precise quantification of the convective and diffusive components of the solute flux leaving the base of the glycocalyx is required. During filtration and reabsorption transients, protein accumulation below the glycocalyx is determined by both convection and diffusion between the base of the glycocalyx and the tissue compartment. Second, the volume of 180 ml predicted to be reabsorbed after 30 min of reabsorption in Figure 4 is much lower than the commonly quoted figure of close to 500 ml in 60 min after a reduction in microvessel pressure (Lanne and Lundvall, 1992). This preliminary result suggests that reabsorption rates greater than 180 ml over 30 min would be difficult to account for if muscle tissue was the primary source of reabsorbed fluid. The series compartment model enables further evaluation of all three observations.

### The Series Compartment Model. Fluid Exchange During a Change in Pressure Over the Same Range (*P* = 20–13) as in the Single Compartment Model

**FIGURE 5:**
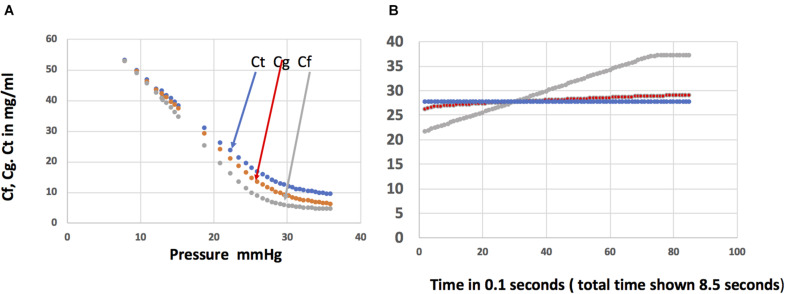
Panel **(A)** shows the steady state concentrations in the fluid in the sub-compartment *Cf* (gray), the sub-compartment between the base of the glycocalyx and the tissue sub-compartment *Cg* (red), and in the tissue sub-compartment *Ct* (blue) as a function of pressure when the barriers behind the glycocalyx account for 7% of the total resistance to exchange across the glycocalyx-endothelial cleft pathway (PdS2 = 5.6 ml/min; PdS1 = 0.4 ml/min). Panel **(B)** shows the time course of changes in the concentrations in each of the three sub-compartments as pressure is reduced in steps of 0.1 mmHg each 0.1 s from a steady state at *P* = 20 mmHg to a pressure of 13 mmHg after 7 seconds. A detailed analysis of the magnitude and direction of diffusion and convective transport of protein fluxes as pressure is reduced is in the text.

Figure 5A show the steady state plasma protein concentrations in the sub-compartments under the glycocalyx, in the cleft, and in the tissue compartment from the series compartment model when the PS product for diffusion between the sub-glycocalyx and the cleft is 5.6 ml/min. Figure 5B shows the transient changes in the concentrations in the sub-glycocalyx compartment, cleft, and tissue compartments as pressure is reduced in steps of 0.1 mmHg each 0.1 s from steady *P* = 20–13. The initial concentration of plasma proteins in each sub-compartment is the steady state at *P* = 20 mmHg (Figure 5A). The concentration under the glycocalyx *Cf* is 21.56 mg/ml (COP 5.4 mmHg); in the cleft (*Cg*), 26 mg/ml, COP 6.7 mmHg) and in the tissue (*Ct*) 28 mg/ml (COP 7.3 mmHg). The tissue steady state concentrations are, as expected, close to those in the single compartment model. However, in contrast to conditions in the single compartment model where gradients between the base of the glycocalyx and the tissue were neglected and the kinetics of fluid exchange is determined only by slowly changing tissue sub-compartment concentrations, the series compartment model demonstrates the significant changes in the concentration in the compartment under the glycocalyx as pressure changes. The example in Figure 5B enables a direct comparison of the osmotic pressure determining the magnitude and direction of water and solute flux in each compartment model with the single tissue compartment model as described below.

#### Filtration *P* = 20–17 mmHg

The key observation is that the protein concentration under the glycocalyx from the moment pressure is first reduced tracks directly with pressure. If pressure had been changed more rapidly, the concentration under the glycocalyx would also have changed more rapidly. *Cf* increases with each 0.1 mmHg pressure reduction because the filtration rate falls below that sufficient to offset back diffusion from cleft compartment (26 mg/ml) versus the glycocalyx sub-compartment (21.6 mg/ml). Solute continues to accumulate by this mechanism as pressure falls. Each step reduction in pressure causes solute to accumulate by reducing the water velocity opposing diffusion toward the glycocalyx.

Figure 5B shows that when pressure reaches 17 mmHg after 3 s, protein concentration in the glycocalyx compartment is very close to that in the cleft compartment and both are very close to the mean ISF concentration, all 28 mg/ml (COP = 7.3). The filtration rate is the same as predicted from the single compartment model. This demonstrates that the assumption in the single tissue model that *Cf* can be set equal to the tissue immediately after a step reduction in pressure is a very reasonable approximation.

#### Reabsorption in the Series Compartment Model: Pressure Reduced From 17 to 13 mmHg

At a pressure of 16.4 mmHg filtration ceases when the net filtration pressure is zero. Reabsorption begins as pressure is reduced further and protein, carried into the cleft from the tissue sub-compartment by solvent drag, accumulates there. The direction of the diffusion gradient (glycocalyx to cleft) reverses (red line crosses the gray line in Figure 5B). When pressure stabilizes at *P* = 13 mmHg, the solute concentration beneath the glycocalyx is 36.8 mg/ml (COP of 10.4 mmHg). This concentration determines the net driving force for reabsorption the moment *P* is 13 mmHg. In contrast to the case during filtration, accumulation causes the concentration of protein under the glycocalyx to be significantly higher than the tissue concentration (36.8 mg/ml vs. 28 mg/ml; COPs 10.4 vs. 7.33 mmHg, respectively). The tissue concentration is not a useful substitute for the protein concentration under the glycocalyx. This is why the single tissue model overestimates the rate of reabsorption.

Our model demonstrates that convective transport of protein into the cleft dominates over back diffusion until the sub-glycocalyx reaches 37.1 mg/ml. At this point back diffusion limits further build-up of protein under the glycocalyx. Thereafter a slow readjustment of the rate of back diffusion determines the time course of reabsorption along with changes in plasma protein concentration. We analyse this example further in the section “Discussion.”

**FIGURE 6:**
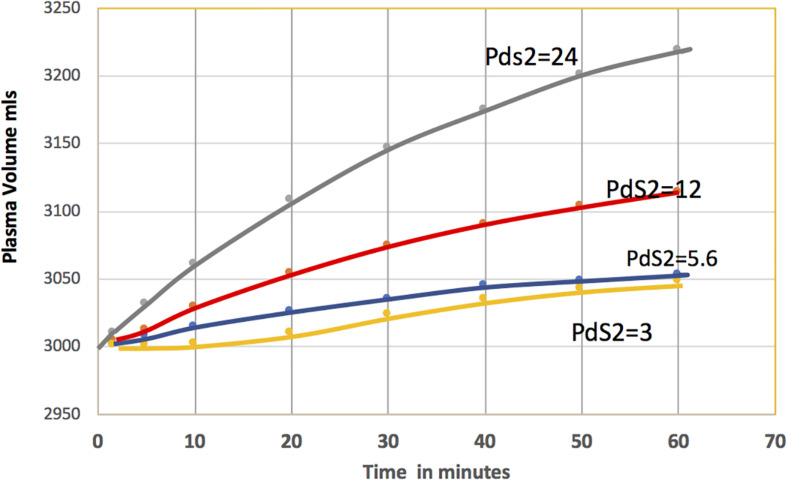
The plasma volume as a function of time is shown for a range of values the PS product for the barrier between the glycocalyx sub-compartment and the sub-compartment in the cleft. The highest resistance to diffusion (10% of total corresponds, PdS2 = 3 ml/min) results in the least volume reabsorbed (orange curve). Reduced resistance increases the volume reabsorbed: 7% of resistance, PdS2 = 5.6 ml/min, blue; 4% of resistance, PdS2 = 12 ml/min, red) and 1% of resistance, PdS2 = 24 ml/min, blue). These results can be compared with Figure 4A where the resistance in the cleft was effectively zero.

The conclusion from the reabsorption case is that the average ISF concentration can significantly underestimate the concentration under the glycocalyx in the presence of a diffusion barrier between the glycocalyx compartment and the tissue compartment. The volume reabsorbed for a range of values for the PS product for the barrier between the glycocalyx and cleft compartment is shown in Figure 6.

The value of PS of 5.6 ml/min assigned to the barrier between the glycocalyx compartment and the cleft In Figure 5 corresponds to diffusion barriers that accounted for 7% of the total transvascular resistance. The blue curve in Figure 6 is the corresponding time course of reabsorption over 60 min. After 30 min, 36 ml are reabsorbed. This is less than 20% of the 180 ml predicted using the single tissue sub-compartment in Figure 4. The lowest curve in Figure 6 shows that the volume is further reduced when these diffusion barriers account for 10% of the resistance (PS = 3 ml/min). On the other hand, decreased resistance increases the volume reabsorbed after 30 min to 117 ml (PS = 12 ml/min, 4% of total resistance). Only a further increase in PS to 24 ml/min (1% of diffusion resistance) predicts a volume reabsorbed of 150 ml in 30 min, approaching that in the single tissue compartment. The increased reabsorption reflects more rapid dissipation of solute accumulation under the glycocalyx. Taken together, Figures 5, 6 provide the first examination of the range of permeability characteristic for barriers between the glycocalyx and the tissue sub-compartment that would support reabsorption when the glycocalyx is present and both the ultrastructure of the junction in a continuous capillary and the anatomy of muscle tissue are considered. They are consistent with the tentative conclusion from the single compartment model that, for the skeletal muscle parameters selected, reabsorption from muscle is unlikely to account for volume up to 500 ml over 60 min.

## Discussion

### Overview

Our results demonstrate the importance of recognizing the small pore glycocalyx as part of a barrier to fluid exchange between blood and the body tissues that includes additional series and parallel pathways for exchange. Barriers to water and solute exchange within the endothelial cleft in series with the glycocalyx determine the extent to which the plasma protein concentration beneath the glycocalyx differs from the protein concentration in the ISF. Furthermore, both steady state and transient exchange across the glycocalyx is modified by the presence of large pores that carry a large proportion of total transvascular solute flux at low capillary pressures. In their simplest form large pores can be understood as regions when the glycocalyx is degraded in microvessels where the inter-endothelial junctions are less tight (e.g., in venular microvessels). While the functions of this combined barrier during steady state filtration are reasonably well understood (Levick and Michel, 2010; Curry, 2018; Michel et al., 2020), our analysis demonstrates a fundamental difference between filtration and reabsorption across the endothelial barrier in the presence of the glycocalyx. As illustrated by transient filtration, new filtration rates after a small change in capillary pressure can be predicted quite accurately from the steady state conditions that exist just prior to the change in pressure. However, as illustrated by examples in Figures 5, 6, prediction of the magnitude and time course of reabsorption rates requires detailed understanding of diffusive and convective transport in the cleft beneath the glycocalyx. Following a pressure drop that results in a change from filtration to reabsorption, there is rapid accumulation of plasma proteins from the ISF beneath the glycocalyx that reduces the reabsorption rate well below that expected from the initial conditions. There is then a prolonged period of gradually reducing reabsorption rates as plasma protein concentrations below the glycocalyx and in tissue approach to a new steady state.

Our modeling is at best a first step toward a more detailed analysis of such reabsorption transients. This is important because the results showing reabsorption of volumes of the order 50–250 mls of ISF from 40 kg of muscle ISF over a period of 30 min would not account for reports in human subjects that up to one half-liter of interstitial fluid can be returned to the plasma volume after a significant fall in capillary pressure. At this time we do not know whether it is valid to use predictions from the model in their present form to make detailed comparisons with observations in human subjects. However, some clinical investigators are currently using models of transvascular flow that fail to consider any of the mechanisms that our approach describes (Hahn, 2020). Additional studies with the models in this paper can help to bridge this gap between purely empirical data fitting models and more mechanistic analyses. To guide such future investigations the section “Discussion” below provides a detailed analysis of the strengths and weakness of the models to identify areas for further investigation.

### Comparison With Previous Models

Within the endothelial glycocalyx and barriers in the inter-cellular cleft there are significant differences in the solute concentration over length scales of tens to hundreds of nanometers. On the other hand, the diffusion distances in the ISF beyond the cleft exit are tens to hundreds of microns and the corresponding concentration gradients can be much smaller than that in the cleft. This makes numerical calculations that accurately account for the wide range of length scales difficult to use. Previously the 3D model used a combination of analytical and numerical methods for a detailed hydrodynamic analysis that accounted for convective-diffusion exchange across the glycocalyx and through an idealized arrangement of the junction strands (Hu et al., 2000; Adamson et al., 2004). Despite the success of this model to account for measured transvascular flows in individually perfused microvessels in the mesentery, it was far too complex to be used for further experimental investigation. A 1D equivalent model was developed to describe convection and diffusion through successive layers of glycocalyx and the junction cleft by choosing transport coefficients for barriers in the cleft so that the transvascular fluxes of water and solute in the 1D model were equivalent to those in the 3D model (Zhang et al., 2006). The modified model was extended to account for the observation that reabsorption transients in individually perfused mesenteric microvessels approaches a new steady state after 2 min, instead of an expected time greater than 1 h (Michel and Phillips, 1987; Zhang et al., 2008).

The series compartment model evaluated in this paper extends this approach, but it is important to stress that this model is far less sophisticated than even the 1D. The simplifying step was to lump the resistance with each successive layer of the 1D model as a membrane with permeability characteristic of the resistance expected to transport into and across that layer. Water and solute flux are assumed to exchange across this membrane into a well-mixed volume with dimensions characteristic of each layer. When reasonable volume estimates are assigned, transient changes are described in terms of the composition of plasma protein within each compartment determined by a simple mass balance over each sub-compartment.

### Series Compartment Model

The most important result from this lumped parameter-series compartment model is the quantitative evaluation of solute accumulation under the glycocalyx during reabsorption. Figure 5 demonstrates one example as pressure was reduced in steps on 0.1 mmHg every 0.1 s. Small time steps were necessary because modeling at a time scale of seconds is incompatible with the length scales in this model. For example a sudden pressure drop from 20 to 13 mmHg with no change in the concentration difference across the glycocalyx would cause such a large reabsorption rate (>10 ml/min) that convective transport of plasma proteins into the cleft opening to the tissue compartment would exceed 300 mg/min (5 mg/s) and the protein concentration in the glycocalyx sub-compartment would exceed plasma protein concentration. This is an example of the problem Weinbaum and colleagues anticipated when setting up the 3D model.

### Convection vs. Diffusion in the Sub-Glycocalyx Compartments

The relative contributions of diffusion and convective transport are determined by the Peclet number when used in the Hertzian equation for transport across a membrane. For exchange between the sub-compartment under the glycocalyx concentration *Cf* and the cleft compartment *Cg*:

(21) $$Js=Jv1\left(1-\sigma \right)\left[\frac{C\,f-C\,g\,e\,x\,p\,\left(-P\,e\right)}{1-\exp\left(-P\,e\right)}\right\}$$

*Pe* is the ratio of the velocity of the solute due to solvent drag to the diffusive velocity over the barrier between the compartments. The convective solute velocity is measured as *Jv* (1 − σ) and the diffusive velocity as *Pd*. This relation can be used to evaluate the relative contribution of convective and diffusive transport at any stage of a transient. For example, at *P* = 13 mm Hg, *Cf* = 36.8 mg/ml and *Cg* is 28.8 mg/ml. Setting the direction *Cf* to *Cg* as positive, the question is whether *Js*1 is directed away from the glycocalyx sub-compartment due to diffusion or into the sub-compartment due to convection.

Equation 21 shows that the magnitude and direction of *Js* depends on the difference between the value of *Cf* and *Cg*exp(-Pe). For *P* = 13 mmHg, the Peclet Number corresponding to the calculated reabsorption rate (1.95 ml/min) is −0.279. The value of *Cf*−*Cg*exp(-Pe) calculated as 36.8–38 mg/ml, is negative. Protein flux is into the sub-glycocalyx space. Thus when pressure is first set at 13 mmHg, convective flux adds solute from the cleft compartment below the glycocalyx. Specifically the convective flux from the cleft is 0.75 mg/0.1 s and the diffusion back into the cleft is 0.057 mg/0.1 s. Our calculations show that solute accumulation continues to a peak value beneath the glycocalyx of 37.17 mg/ml. The reabsorption rate is then 1.71 ml/min and Pe = −0.244. Now *Cf*−*Cg*exp(-Pe) is positive, and net diffusion is directed away from the glycocalyx. The fine balance between the convective transport into the sub-compartment and diffusion determines the subsequent time course of the reabsorption until reabsorption stops and eventually *Cg* rises as the tissue concentration approaches a new steady state.

It might be argued that the fine balance between back diffusion and convective transport into the sub-compartment below the glycocalyx during reabsorption can be compromised because water velocity increases as the reabsorbed fluid is funneled into the breaks in the junction strand. Although water velocity would indeed increase due to such funneling, this does not mean that convective transport necessarily dominates diffusion at the breaks. This is because the geometry of the break is such that the diffusion distances (Δ*X* strand) corresponding to the region near the breaks where the funneling of reabsorbed fluid occurs are less than 100 nm, relative to total diffusion distances in the cleft (up to 1 μ or 1,000 nm). Thus if breaks in the junction strand (representing 10% of the length of junction strand available to flow) increased local water velocity 10-fold, the local diffusion velocity measured as *D*/Δ*X* strand would also be increased, independently, by a similar order of magnitude (where *D* = diffusion coefficient of protein). The ratio of water velocity to diffusion velocity would remain close to that estimated for the lumped parameters.

### Comparing the Series Compartment Model With the Simple Compartment Model

The assumption used in the single tissue compartment (Figures 1A, 2) is that the concentration in the tissue compartment can be substituted for the sub-glycocalyx concentration during both filtration and reabsorption. As shown by Figure 5, during filtration the tissue concentration is a good approximation to sub-glycocalyx concentration because of the rapid readjustment of concentration gradient under the glycocalyx. A similar rapid adjustment would take place when pressure was increased. This assumption enables a reasonable prediction of volume shifts that occur over periods of tens of minutes because change in tissue concentration toward a true new tissue concentration take place over time periods of 100s of minutes. One example of a practical use of the single compartment model to describe changes in filtration after plasma proteins are diluted by saline infusion has been published (Michel et al., 2020).

On the other hand during reabsorption, the use of the prior steady state tissue concentration as an estimate of the sub-glycocalyx significantly overestimates the driving forces for reabsorption and overestimates the volume reabsorbed. Prior knowledge of the barriers to solute exchange between the base of the glycocalyx and the tissue is required for more accurate evaluation of such fluid exchange. One version of this observation has been recognized for one and a quarter centuries. Transvascular exchange described by the Starling principle is self-limiting. Reabsorption concentrates the plasma protein concentration in the ISF and the corresponding increase in tissue COP limits further reabsorption. This action is amplified many times when reabsorption leads to accumulation of plasma proteins in the much more limited space under the glycocalyx.

### The Composition of the Tissue Compartment

There are many limitations to the treatment of sub-compartments in the ISF. We estimated the time for protein concentration in the smaller endomysial ISF compartment to reach a new steady after a change in pressure to be over 1,000 min when all the protein flux mixed with this compartment. However, as Figure 2 shows, a significant fraction of the large pore flux from venular vessels may exchange directly into the larger perimysium and the time to reach steady state would be corresponding longer. Two additional issues related to tissue composition. One is that the use of an average tissue composition to estimate effective transvascular COP differences ignores the fact that in the steady state the filtration rate and the COP opposing filtration vary as pressure falls from the arterial to the venular side of the microvascular bed. Thus as Levick (1991) demonstrated, the use of average tissue composition predicts filtration rates far in excess of measured lymph flows. A subtler but important second issue is that the composition of the ultrafiltrate from the glycocalyx contains fewer proteins than the plasma and more albumin per unit volume. For a given protein concentration the COP of an ISF sample is expected to be larger than that calculated from Eq. 8 for a plasma sample. The analysis of these interactions requires a far more sophisticated model than considered here.

### Transient vs. Sustained Reabsorption

Zhang et al. (2006) noted that there could be sustained reabsorption in microvessels when capillary pressures were reduced below 17 mmHg when the COP in blood was set to 25 mmHg and the tissue plasma protein concentrations were set at 40% of plasma concentration some distance from the microvessel wall. It is important to emphasize that this result does not contradict the “no steady state reabsorption” rule established by the Revised Starling principle in tissues where the ISF is generated initially as a capillary ultrafiltrate as in Figure 3. The reason these investigators described sustained reabsorption is that the ISF concentration of protein was held constant at 40% of plasma concentrations at all capillary pressures, including those at 17 mmHg and below. In contrast, true steady state tissue concentration increases significantly above 40% plasma concentration at pressures below 17 mmHg when the steady state rule that *Ct* = *Js*/*Jv* summed over all pathways is applied. This observation focuses attention on the rate of change of tissue concentrations after a reabsorption transient as a determinant of the reabsorption transient. We show (e.g., Figure 4) that the time course of reabsorption can be very long in muscle, even when the volume of the sub-compartment of ISF is less than 20% of the total ISF (1,000 ml vs. 6–8,000 ml). When reabsorption begins after steady state filtration at a pressure of 20 mmHg, the tissue plasma concentration is in the range of 30–40% blood concentration as in Figure 3D. After a rapid re-adjustment of concentration beneath the glycocalyx as pressure falls there is slow reabsorption for more than an hour because tissue concentration increases slowly. This is similar to that described by Zhang et al. (2006) with a fixed tissue concentration.

It is also noted that there can be a clear exception to “no sustained reabsorption” rule. In the kidney, lymph nodes, and absorbing GI tissues there are additional mechanisms to limit the extent of plasma protein concentration in the tissue and a compartment beneath the glycocalyx (especially in fenestrated capillaries as described elsewhere; Michel et al., 2020). These mechanisms to maintain steady state reabsorption are not present in muscle.

### Other Junction Configurations and the Sensitivity of the Models to Uncertainty in the Parameters

Our calculations emphasize the need to understand the resistance to water and solute transport between the base of the glycocalyx and ISF in a local tissue compartment. Freeze fracture images of the intercellular cleft reveal multiple junction strands within the junction of capillaries of diaphragm and heart muscle (Wissig and Williams, 1978; Bundgaard, 1984). In contrast to the simple geometry for strands used in the present analysis, the tortuous pathway though the junction complex suggests a resistance to both water and plasma protein higher than in the present analysis. When setting up the 3D model, Hu et al. (2000) argued that the distributed resistance of multi strand patterns in mesenteric microvessel to diffusion could be replaced by the single equivalent barrier, but the validity of this argument for more complex multi-layer arrays for both solute and water flows remains to be evaluated. This is important because the predictions of protein concentrations across the glycocalyx are most sensitive to uncertainties in the volumes of the sub-compartments *Vf* and *Vg* within the cleft, and to the diffusion resistance to exchange between *Vf* and *Vg* as illustrated in Figure 6. Furthermore, a notable omission in the present analysis is consideration of resistance to water flow in the space under the glycocalyx. A better description of the pressure differences between the space under the glycocalyx and the tissue compartment is needed.

### Other Model Parameters

We have considered only the limiting case where the large pore pathway mixed with the endomysial compartment. If exchange occurred between the endomysial and the perimysium the effective volume regulating tissue concentration would increase. Further we have previously estimated that there is, on average, only one to two large pores per microvessel in tissues such as muscle (Michel and Curry, 1999). It is not unreasonable to suggest that some regions of the microvascular bed have no large pores. There is limited understanding of the extent that fluid and solute exchange into and between the endomysial and the perimysial compartments over time periods of 30–60 min. Our estimates of the total volume of ISF fluid reabsorbed after reduction in capillary pressure would be underestimated if the volume used for the tissue compartment was too small or tissue plasma protein concentrations were lower than the steady state concentrations we used. At the same time it should be noted that clinical and experimental measurement of changes in plasma volume based on small changes in hematocrit are prone to much larger errors than are commonly recognized (Michel and Curry, 2009). Progress toward better understanding of the way sub compartments of the ISF modulate transvascular fluid exchange in whole organs such as the skeletal muscle mass in human subjects requires refinements in both the models described in this paper and the measurement of changes in plasma volume in human subjects during filtration and reabsorption.

### Summary

The primary purpose of these investigations is to integrate our growing knowledge about the endothelial glycocalyx as a permeability and osmotic barrier into models of trans-vascular exchange in whole organs. For plasma proteins we describe changes in their COP difference across the glycocalyx after an increase or decrease in capillary pressure. The composition of the fluid under the glycocalyx can change in step with capillary pressure, whereas the average composition of the interstitial fluid takes many hours to adjust to the same change in vascular pressure. We used models where the fluid under the glycocalyx mixes with sub-compartments of the ISF whose volumes could be reasonably well defined from the ultrastructure of the inter-endothelial cleft and the histology of the tissue surrounding the capillaries. The initial protein composition in the sub-compartment is set as that during steady state filtration in the presence of a large pore pathway in parallel with the “small pore” glycocalyx pathway. Changes in the composition of the sub-compartments depend on their volume and the balance of convective and diffusive transport into and out of each compartment. We used skeletal muscle as an example. The simplest model assumes the fluid under the glycocalyx mixes directly with a tissue sub-compartment with a volume less than 20% of the total skeletal muscle ISF volume. This model places limits on transvascular flows during transient filtration and reabsorption over periods up to 60 min. The key assumption in this model is compromised when the resistance to diffusion between the base of the glycocalyx and the tissue volume accounts for more than 1% of the total resistance to diffusion across the endothelial barrier formed by the glycocalyx and intercellular cleft. It is well established that in steady state, the balance of hydrostatic and colloid osmotic forces across the glycocalyx requires that reabsorption does not occur in tissue such as skeletal muscle. Our approach extends this idea to demonstrate that the change in vascular pressure favoring transient reabsorption from the skeletal muscle interstitial fluid result in much less fluid exchange than is commonly assumed.

We envision at least two further extensions of the approach used in the present analysis. One is to apply a similar analysis to other organs where reasonable estimates of capillary ultrastructure and ISF histology is available. The lung would be of particular interest as would the G-I tract because, with its large fraction of resing cardiac output and permeable capillaries, it could have large and rapid effects on plasma composition. The second is to enable critical evaluation of the empirical models of trans-vascular fluid exchange being used for in some clinical applications. While there is an understandable desire to describe fluid exchange between plasma and tissue as simply as possible, the balance of hydrostatic and COPs across the glycocalyx cannot be ignored. This is the case in the approach known as “volume kinetic analysis” which describes fluid exchange between the plasma compartment and an arbitrarily defined tissue sub-compartment assuming fluid exchange is proportional to the changes in the volume of fluid in these compartments from an initial state (Hahn, 2020). The failure of these models to account for the fine balance of hydrostatic and COPs across the glycocalyx that our model describes must compromise their utility.

## Data Availability Statement

The original contributions presented in the study are included in the article/supplementary material, further inquiries can be directed to the corresponding author.

## Author Contributions

FC drafted the manuscript. Both authors discussed the changes that lead to the final version.

## Conflict of Interest

The authors declare that the research was conducted in the absence of any commercial or financial relationships that could be construed as a potential conflict of interest.

## Publisher’s Note

All claims expressed in this article are solely those of the authors and do not necessarily represent those of their affiliated organizations, or those of the publisher, the editors and the reviewers. Any product that may be evaluated in this article, or claim that may be made by its manufacturer, is not guaranteed or endorsed by the publisher.
